# Altered EEG microstate dynamics in adolescents with non-suicidal self-injury at rest and following acute social exclusion

**DOI:** 10.3389/fpsyt.2026.1853860

**Published:** 2026-07-16

**Authors:** Congcong Liu, Yajing Si, Yining Kou, Huili Xing, Xin Wu, Bin Shi, Liju Qian, Kun Li, Meng Zhang

**Affiliations:** 1 Department of Psychology, Henan Medical University, Xinxiang, Henan, China; 2 Institute of Brain and Psychological Sciences, Sichuan Normal University, Chengdu, Sichuan, China; 3 School of Education, Shanghai Normal University, Shanghai, China; 4 Department of Psychology, Hangzhou Normal University, Hangzhou, Zhejiang, China; 5 Shandong Daizhuang Hospital, Jining, Shandong, China; 6 Jining Key Laboratory of Neuromodulation, Jining, Shandong, China

**Keywords:** adolescents, electroencephalography, microstate analysis, non-suicidal self-injury, social exclusion

## Abstract

**Background:**

Non-suicidal self-injury (NSSI) is highly prevalent in adolescence, yet the fast dynamics of large-scale brain networks remain poorly understood. We aimed to characterize resting-state EEG microstate dynamics in a clinically recruited adolescent NSSI sample and to examine state-dependent changes following acute social exclusion.

**Methods:**

Resting-state EEG was recorded in hospitalized, medicated adolescents with NSSI and in healthy controls (HCs). An NSSI subgroup then completed EEG assessments before and after either Cyberball-induced social exclusion or a non-stress control condition. Microstate parameters and transition probabilities were analyzed.

**Results:**

After multiple-comparison correction, the core baseline finding was robustly reduced microstate A duration in the NSSI group relative to HCs. In uncorrected exploratory analyses, additional differences included shorter durations of microstates B and F, higher occurrence of microstate D, and increased F→D transition probability. A multivariate pattern analysis suggested group-related information but must be interpreted cautiously given the modest sample size. Within the NSSI group, the exclusion group showed reduced microstate A expression and increased microstate D expression relative to the non-stress subgroup, with a broader transition shift toward microstate D. Several interaction effects survived correction.

**Conclusions:**

In this medicated inpatient sample, resting-state microstate dynamics differed from HCs, most robustly in reduced microstate A duration, and reorganized after social exclusion. Specificity to NSSI remains uncertain given the absence of an MDD-only clinical-control group and healthy Cyberball comparison. These preliminary findings require replication in larger studies including medication-naive MDD-only, MDD+NSSI, and HC groups.

## Introduction

Non-suicidal self-injury (NSSI) refers to the deliberate destruction of one’s own body tissue without suicidal intent and for purposes not socially sanctioned (1). It is particularly prevalent during adolescence and young adulthood, with rates in Chinese adolescent samples reported to be as high as 12.2% (2). NSSI is associated with a broad range of adverse outcomes, including depression, anxiety, and elevated risk of later suicidal thoughts and behaviors (3–5). Accordingly, NSSI represents an important clinical and public health concern.

Contemporary models propose that NSSI often serves an emotion-regulatory function (6, 7). Individuals commonly report engaging in NSSI to reduce intense negative affect, interrupt dissociative states, or enact self-punishment (7–10). From this perspective, vulnerability to NSSI may be linked to dysfunction in neural systems involved in emotion processing, self-referential experience, and cognitive control. Functional MRI studies have identified abnormalities in these systems, including altered connectivity involving the amygdala and prefrontal cortex (11). However, because the psychological states preceding NSSI may be transient and rapidly fluctuating, methods with higher temporal resolution are needed to characterize their underlying dynamics.

EEG microstate analysis provides a useful framework for examining the fast dynamics of large-scale brain networks (12–14). In this approach, the continuously evolving scalp electric field is represented as a sequence of quasi-stable topographical configurations, known as microstates (13, 14). These microstates have been proposed to reflect brief, coordinated states of large-scale neural activity and are often described as the “atoms of thought” (15, 16). Across individuals and age groups, four canonical microstate classes (A–D) have been consistently identified, with converging evidence linking them to partially distinct functional systems, including phonological or auditory processing (microstate A), visual processing (microstate B), salience/interoceptive processing (microstate C), and the dorsal attention network (microstate D) (17–23). In addition to the four canonical microstates, a growing number of studies have reported an additional microstate topography (variably labeled E or F) that has been suggested to be part of the Default Mode Network (DMN) and plays a role in personally significant information processing, mental simulations, and theory of mind (18). Altered DMN dynamics have been implicated in emotion dysregulation and self-referential processing deficits observed in adolescents with NSSI.

Alterations in microstate temporal dynamics, including duration, occurrence, coverage, and transition probabilities, have emerged as sensitive indicators of large-scale network dysfunction in psychiatric disorders such as major depressive disorder and generalized anxiety disorder (24–26). Given the close association of NSSI with these conditions, as well as its strong links to emotion dysregulation (27), it is plausible that adolescents with NSSI may also exhibit characteristic microstate abnormalities. Preliminary evidence supports this possibility: a recent study identified distinctive microstate patterns in depressed adolescents with versus without NSSI (28). Several recent EEG microstate studies have attempted to clarify this issue by comparing MDD-only, MDD+NSSI, and HC adolescents. These studies have reported partially different patterns involving microstate A, microstate B, and transition dynamics, suggesting that NSSI-related neurophysiological alterations may partly overlap with depression-related large-scale network abnormalities. Therefore, studies of NSSI should be interpreted in relation to depressive psychopathology and transdiagnostic emotion-dysregulation mechanisms. However, it remains unclear whether NSSI is associated with a distinguishable resting-state neurodynamic profile in its own right, and how such a profile may respond to ecologically relevant interpersonal stressors.

Acute psychosocial stress, particularly social exclusion and interpersonal rejection, is a well-established proximal trigger of NSSI urges and behaviors (29–32). Understanding how vulnerable adolescents respond neurophysiologically to such stressors is therefore important for clarifying the proximal mechanisms that may precipitate self-injury. The Cyberball paradigm is a well-validated experimental task for inducing social exclusion in a controlled setting (33). Examining microstate dynamics before and after such exclusion may provide insight into the rapid neural reconfiguration associated with acute interpersonal distress.

The present study had three aims. First, we sought to characterize resting-state EEG microstate dynamics in a clinically recruited, hospitalized adolescent NSSI sample relative to healthy controls, with the goal of describing neurophysiological features associated with this clinical population. Second, we examined whether distributed microstate features contained information that could separate the NSSI and healthy control groups within an exploratory multivariate pattern analysis framework. Third, we investigated whether acute social exclusion, induced by the Cyberball paradigm, was followed by changes in microstate dynamics within the NSSI group, in order to describe state-dependent neurophysiological reorganization following interpersonal stress. We hypothesized that microstates related to self-referential processing and attention would show group-related differences at baseline and condition-related changes after social exclusion. All analyses were regarded as exploratory and descriptive given the modest sample size, the absence of a clinical-control group, and the medicated status of the NSSI participants.

## Method

### Participants

The sample size was not determined by an *a priori* power analysis because no directly comparable EEG microstate study combining adolescent NSSI, healthy controls, and Cyberball-induced social exclusion was available to provide reliable effect-size estimates. Recruitment was therefore based on clinical feasibility and on sample sizes used in related adolescent EEG microstate studies of MDD/NSSI and adolescent depression (26, 28, 34, 35). A total of 39 adolescents aged 11–18 years with NSSI was recruited from Shandong Daizhuang Hospital through advertisements posted in inpatient departments. All NSSI participants was hospitalized and receiving inpatient pharmacological treatment. Detailed medication records (including specific compounds, dosages, and duration) were not available for this study; therefore, the potential influence of psychotropic medications on EEG microstate parameters could not be assessed. NSSI diagnosis was confirmed by experienced psychiatrists according to DSM-5 diagnostic criteria for non-suicidal self-injury and further supported using a previously validated self-report measure (36). Inclusion criteria for the NSSI group were: (1) at least five days of intentional self-inflicted damage to body tissue without suicidal intent during the past year; (2) right-handedness; and (3) ability to understand the study procedures. Exclusion criteria included current or past schizophrenia spectrum disorders, pervasive developmental disorders, medical or neurological diseases, or psychiatric disorders secondary to a general medical condition. Standardized quantitative measures of NSSI frequency, recency, severity, functions, urges, and duration of NSSI history were not systematically collected as part of the research protocol; therefore, clinical heterogeneity within the NSSI group and its potential influence on EEG microstate dynamics could not be examined. A total of 32 age-matched healthy controls (HCs) were also recruited. HC participants were required to have no current or past psychiatric diagnosis and no history of NSSI. The study was approved by the Ethics Committee of Shandong Daizhuang Hospital (Approval No. 202411KS-1). Written informed consent was obtained from all participants and their legal guardians before participation.

Of the 39 adolescents initially recruited into the NSSI group, four were excluded at baseline (withdrawal, n = 1; not meeting NSSI criteria, n = 2; suicide attempt within the previous week, n = 1). The final baseline sample therefore included 35 adolescents with NSSI and 32 HCs. Of the 35 participants with NSSI, 20 were assigned to the acute social exclusion group and 15 to the non-stress control group. For the post-manipulation EEG analysis, data from three NSSI participants were excluded because of excessive EEG artefacts, resulting in final samples of 19 participants in the acute social exclusion group and 13 participants in the non-stress control group.

### Acute social exclusion manipulation

**Figure 1:**
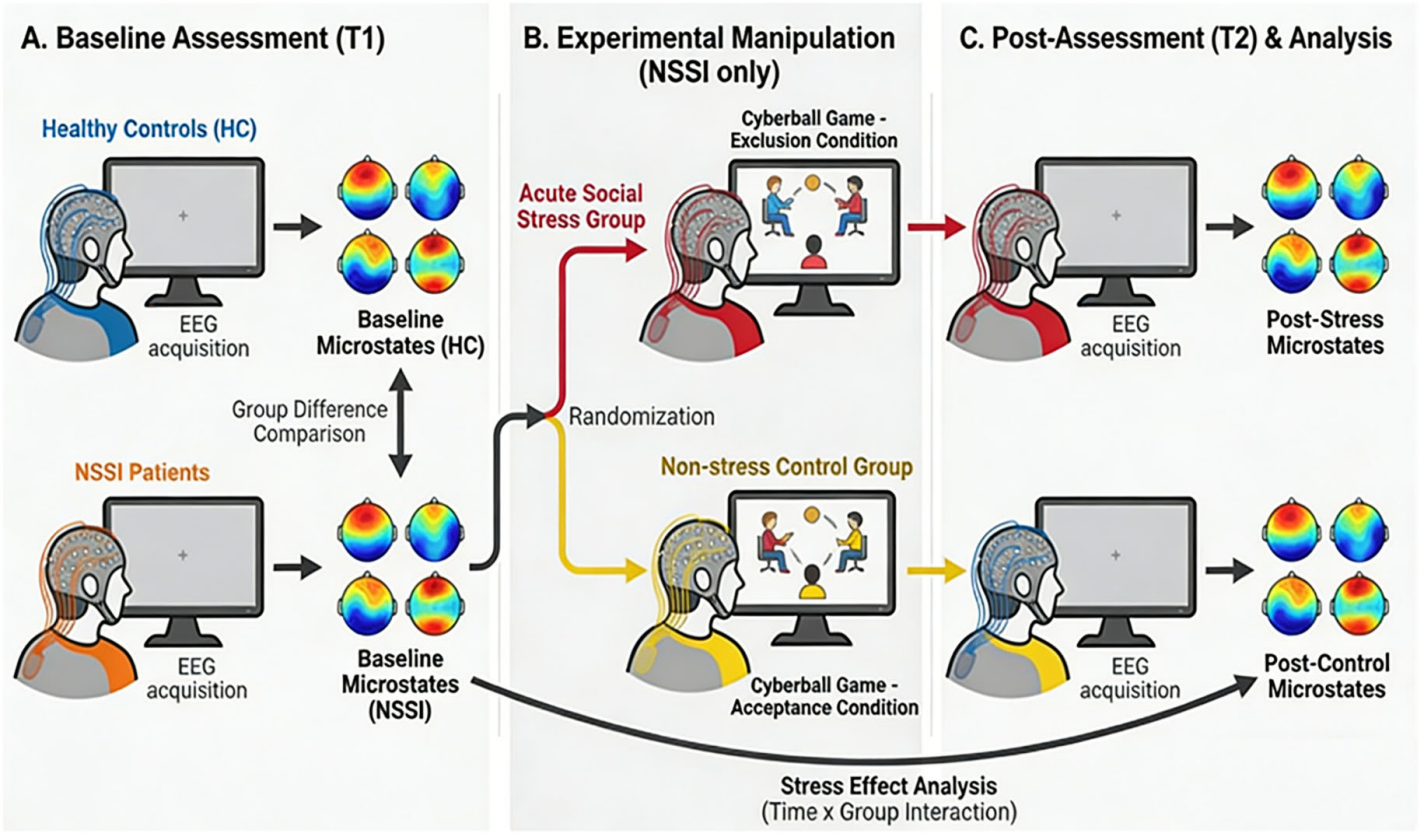
Cyberball paradigm and experimental procedure. NSSI, Non-suicidal self-injury; T, Timepoint.

The Cyberball task, a virtual ball-tossing game, was used to induce acute social exclusion (**Figure 1**). In this paradigm, participants believed that they were playing with two other individuals, although the other “players” were controlled by a computer program. In the non-stress control condition, they received approximately one-third of all ball tosses (10 of 30 throws). In the acute social exclusion condition, the participant received only two throws initially and was then excluded.

During the instruction phase, participants were told that the task was designed to assess mental imagery ability and were asked to imagine the situation vividly. The task lasted approximately 5 min. Immediately afterward, standardized verbal questions assessed subjective experiences (e.g., exclusion, distress); responses were documented as a procedural check to confirm manipulation face validity, not as a formal outcome measure.

### Procedure

Upon arrival at the laboratory, participants were given a 5-min adaptation period to acclimatize to the testing environment. They then prepared for EEG recording by washing and drying their hair. After assessment of clinical covariates, baseline resting-state EEG was recorded while participants sat quietly with their eyes open.

Participants with NSSI were then randomly assigned to either the acute social exclusion group or the non-stress control group. After completion of the Cyberball task, resting-state EEG was recorded again (**Figure 1**).

### Clinical measures

To characterize depressive and anxiety symptoms, the Hamilton Depression Rating Scale (HAMD) and Hamilton Anxiety Rating Scale (HAMA) were administered by experienced psychiatrists.

### EEG acquisition

EEG data were recorded using a 64-channel wireless EEG system (NeuSen W series, Neuracle Technology Co., Ltd.) with electrodes positioned according to the international 10–20 system. Conductive gel was applied to maintain electrode impedance below 5 kΩ. Recordings were obtained in a quiet, temperature-controlled room. Participants were instructed to remain awake, relaxed, and to keep their eyes open throughout the recording. Resting-state EEG was recorded with a marker denoting the onset of the quiet resting period and a second marker at exactly 5 minutes. Recording continued for an additional 3–5 minutes beyond the 5-minute mark to ensure sufficient artifact-free data for segment selection, resulting in total recording durations of 8–10 minutes.

### Pre-processing of EEG data

EEG preprocessing was conducted in Python using the MNE-Python, pyprep, and Autoreject packages (35). For each participant, a fixed 300-second segment was selected from the recording through visual inspection conducted by two researchers who reached consensus. Irrelevant channels were removed (EOG, M1, M2, P1, and P2). Next, a one-pass zero-phase non-causal finite impulse response (FIR) filter was applied to band-pass the data between 2 and 20 Hz (36). This frequency range was selected to optimize the signal-to-noise ratio for canonical microstate topographies (13) and to minimize contamination from muscle artifacts (37). The filtered data were then downsampled to 250 Hz.

Bad channels were identified via visual inspection and RANSAC algorithm, then interpolated (38). The data were then re-referenced to the common average. Preprocessed signals were segmented into 2-s epochs. Extended Infomax independent component analysis (ICA) was applied to remove artifactual components related to eye movements, electrocardiographic activity, and electromyographic activity (39). Autoreject optimized peak-to-peak rejection threshold using a combination of cross-validation, robust error metrics, and Bayesian optimization. Participants with more than 20% bad epochs were excluded. All remaining participants contributed more than 4 min of valid EEG data after preprocessing.

### Microstate analysis

Microstate analysis was conducted using Pycrostates (40). For each participant, global field power (GFP) was computed as the standard deviation of sensor voltages across electrodes at each time point, and the minimum distance between consecutive GFP peaks was set to 12 ms to ensure robust peak selection. GFP local maxima were used because they correspond to moments with the highest signal-to-noise ratio (41).

To improve clustering stability, microstate segmentation was performed in two stages. First, individual-level clustering was conducted. For each participant, GFP peaks were resampled 20 times (3,000 samples per iteration), and polarity-invariant modified k-means clustering was applied to solutions ranging from 3 to 7 microstate classes. Clustering quality was evaluated using a composite criterion based on the Silhouette score, Calinski–Harabasz score, Dunn score, and Davies–Bouldin score (42–44). These criteria were used to determine the optimal number of clusters for each resampled dataset.Second, group-level clustering was performed by aggregating the individual clustering solutions and reclustering them to obtain five group-level microstate classes, labelled A, B, C, D, and F. Spatial correlation analysis of the resulting topographies across the four groups showed correlations above 70% for corresponding classes, supporting the comparability of subsequent parameter estimates.

The resulting maps were back-fitted to each participant’s preprocessed EEG data by assigning each time point to the template with the highest absolute spatial Pearson correlation coefficient. To mitigate noise-driven transitions, temporal smoothing was applied with a minimum segment duration threshold of 32 ms (corresponding to 8 timepoints at 250 Hz). This threshold was selected based on neurophysiological evidence indicating that meaningful microstates typically persist for 60–120 ms (13, 45). Segments shorter than 32 ms were removed and reassigned to adjacent segments to reduce the influence of transient, non-representative activations. Finally, the following microstate parameters were computed for each participant: global explained variance (GEV), time coverage, mean duration, occurrence rate, and transition probabilities.

### Exploratory multivariate pattern analysis

To examine whether distributed EEG microstate features contained information that could separate adolescents with NSSI from HCs, we implemented an exploratory multivariate pattern analysis based on 35 baseline microstate features. To reduce feature redundancy and optimize model performance, we used a hybrid feature-selection strategy that combined statistically significant group-difference features with top-ranked features identified by the Relief-F algorithm (46).

To minimize information leakage and overfitting, model construction and evaluation were performed using a nested cross-validation framework (47). The outer loop consisted of 5-fold stratified cross-validation for performance estimation, whereas the inner loop used 3-fold stratified cross-validation for hyperparameter tuning of the support vector machine (SVM) model (details in the **Supplementary Materials**). Before model fitting, features were standardized using Z-score normalization. Classification performance was evaluated using accuracy, precision, recall, F1-score, and area under the receiver operating characteristic curve (AUC). Bias-corrected and accelerated (BCa) bootstrap resampling (1,000 iterations) was used to estimate 95% confidence intervals for accuracy and AUC. All feature selection and standardization procedures were performed within the training data of each cross-validation fold only. Given the limited sample size, a permutation test (1,000 iterations) was conducted to assess whether the observed classification performance exceeded chance level (details in the **Supplementary Materials**). Because no independent external validation cohort was available, this analysis was intended as an exploratory internal cross-validation analysis rather than as a clinically validated diagnostic model.

### Statistical analysis

Statistical analyses were conducted using JASP. Demographic and clinical differences between the NSSI and HC groups at baseline were examined using chi-square tests for categorical variables (e.g., sex) and independent-samples t-tests for continuous variables (e.g., age, years of education, and clinical scale scores). Baseline differences between the acute social exclusion and non-stress control subgroups were examined using the same approach, as appropriate.

Independent-samples *t*-tests were used to assess group differences in baseline microstate parameters between the NSSI and HC groups. To control for multiple testing, false discovery rate (FDR) correction was applied within two predefined families of baseline analyses (1): a core parameter family comprising duration, occurrence, and coverage for each of the five microstates (15 tests), and (2) a transition probability family comprising the 20 directional transition probabilities. Effects that survived FDR correction (*p*_FDR_ < 0.05) were described as robust, whereas effects that were significant only before correction were retained as nominal findings and interpreted as exploratory.

To examine the effects of acute social exclusion on microstate dynamics within the NSSI sample, mixed-design analyses of variance (ANOVAs) were conducted with group (acute social exclusion vs. non-stress control) as the between-subject factor and time (pre vs. post) as the within-subject factor. Greenhouse–Geisser corrections were applied where appropriate. FDR correction was applied within the same parameter families as the baseline analyses. Significant interaction effects were followed by simple-effects analyses, with Bonferroni correction applied to *post hoc* comparisons.

For the analytical robustness, a Bayesian repeated measures ANOVA approach was used to provide probabilistic statements for dependent variables that showed significant interaction effects in the mixed ANOVA analyses (details in the **Supplementary Materials**).

## Results

**Figure 2:**
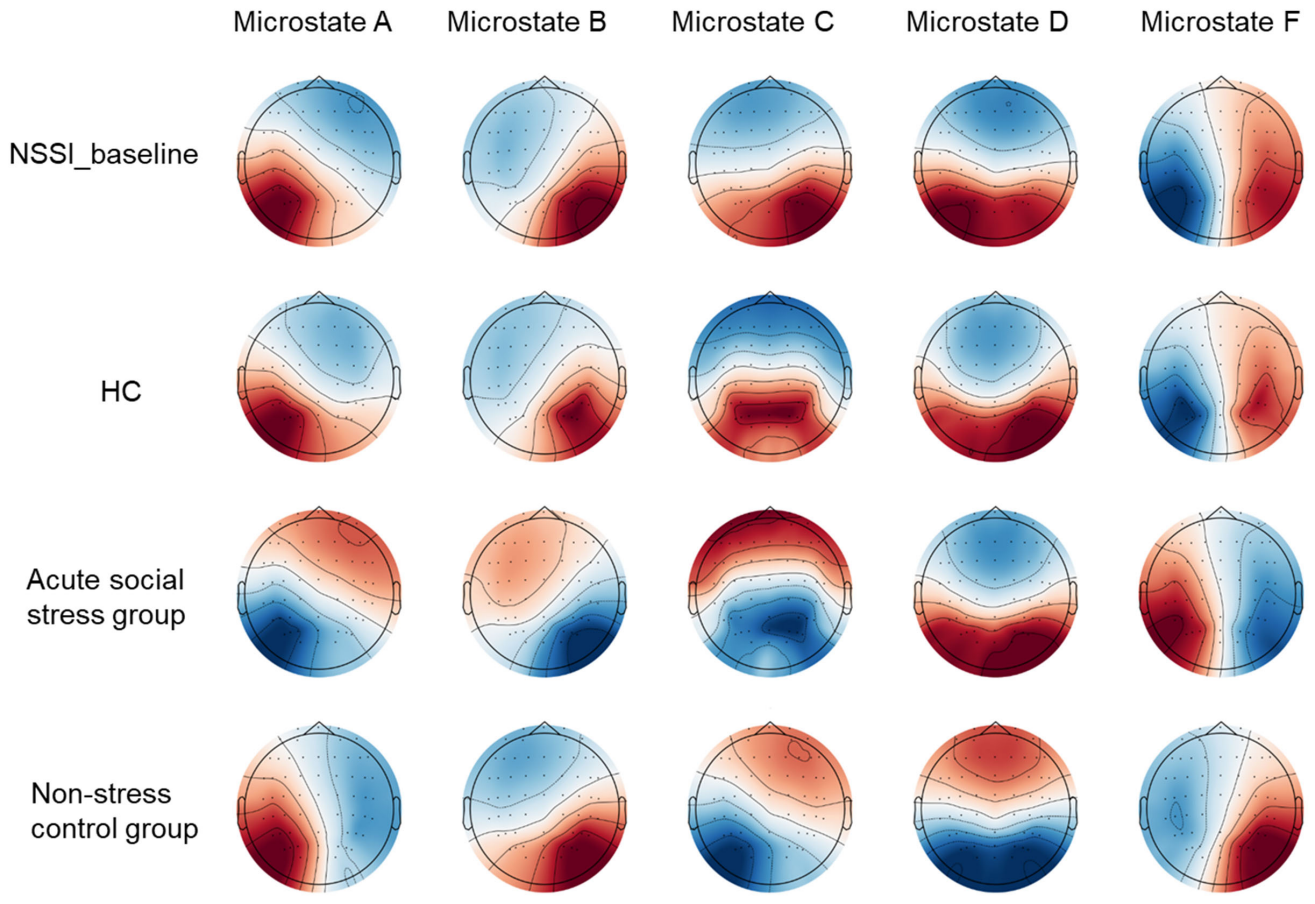
EEG microstate topographies in the NSSI baseline group, healthy controls, acute social exclusion group, and non-stress control group.

As shown in **Table 1**, the NSSI and HC groups did not differ significantly in sex, age, or years of education (all *p*s > 0.05). Likewise, the acute social exclusion and non-stress control subgroups within the NSSI sample did not differ significantly in sex, age, years of education, or clinical scale scores (all *p*s > 0.05). Group-level clustering identified five microstate classes (A, B, C, D, and F) across participants (**Figure 2**). At baseline, global explained variance (GEV) was 63.07% in the HC group and 63.52% in the NSSI group. After the Cyberball task, GEV was 63.48% in the acute social exclusion group and 63.90% in the non-stress control group. These values indicate a modest but descriptively comparable model fit across groups and experimental conditions.

**Table 1:** Demographic and clinical characteristics of the participants.

	NSSI(n=35)		HC (n=32)	t/χ2	p
Variables	Acute social exclusion (n=20)	Non-stress control (n=15)			
Gender(M/F)	5/15	3/12	13/19	2.45	0.117
Age	14.2(1.88)	14.53(2.10)	14.4 (0.62)	0.18	0.856
Education (years)	8.38(1.39)	8.20(1.75)	8.41(0.62)	0.31	0.747
HAMD	13.89(7.02)	13.92(6.35)		0.01	0.993
HAMA	9.11(4.7)	7.75(3.57)		0.85	0.400
Self-harm type					
Scratching	100%	100%			
Hitting	10%	6.67%			
Ingesting non-toxic substances	0	20%			

HAMD, Hamilton Depression Scale; HAMA, Hamilton Anxiety Scale; Comparisons for gender, age, and education years were performed between the HC group and the combined NSSI group (acute social exclusion + non-stress control). Comparisons for HAMD, and HAMA were performed between the two NSSI subgroups only. Note: Self-harm information reported in this table reflects available descriptive data collected during clinical interview and was not derived from a standardized comprehensive NSSI phenotyping instrument.

### Comparison of baseline microstate duration, occurrence, and coverage between the NSSI and HC groups

**Figure 3:**
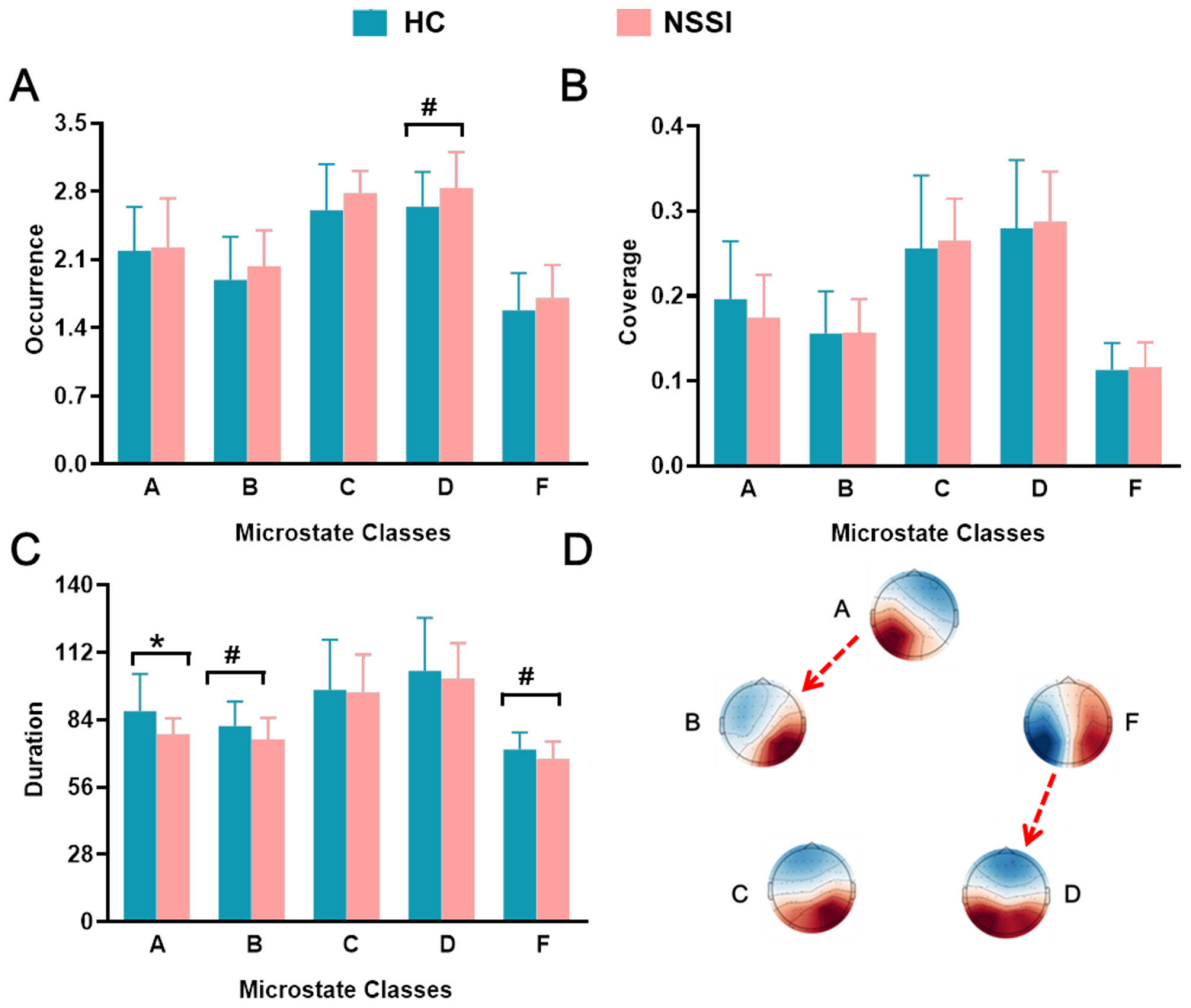
Group differences in EEG microstate parameters between the NSSI and HC groups. Mean values and standard deviations are shown for the HC group (blue, n = 32) and the NSSI group (pink, n = 35). **(A)** Occurrence rate of microstate classes A–D and F. **(B)** Time coverage of microstate classes A–D and F. **(C)** Mean duration of microstate classes A–D and F. **(D)** Transition probabilities showing nominally significant differences between groups. Red dashed arrows indicate transition probabilities that were nominally higher in the NSSI group than in the HC group and did not survive. *p_FDR_ < 0.05; #*p* < 0.05 (uncorrected, did not survive FDR correction).

The core corrected baseline finding was a significantly reduced mean duration of microstate A in the NSSI group relative to HCs, which survived FDR correction (*t*_65_ = 3.35, *p* = 0.001, *p*_FDR_ = 0.015, Cohen’s *d* = 0.81; **Supplementary Table 1**; **Figure 3**). In uncorrected exploratory analyses, the NSSI group additionally showed shorter mean durations of microstates B (*t*_65_ = 2.41, *p* = 0.019, *p*_FDR_ = 0.131, Cohen’s *d* = 0.59), and F (*t*_65_ = 2.23, *p* = 0.029, *p*_FDR_ = 0.131, Cohen’s *d* = 0.55), a higher occurrence of microstate D (*t*_65_ = 2.15, *p* = 0.035, *p*_FDR_ = 0.131, Cohen’s *d* = 0.52). These latter effects did not remain significant after FDR correction and should be regarded as nominal. No significant between-group differences were observed for microstate coverage.

To examine the robustness of the core corrected finding, we performed a univariate analysis of covariance (ANCOVA) with age, gender, and education as covariates. The reduction in microstate A duration remained significant (*F*(1, 62) = 14.11, *p* < 0.001, *η_p_*^2^ = 0.185). As sensitivity analyses for the nominal exploratory findings, ANCOVAs were also conducted for duration of microstates B (*F*(1, 62) = 4.46, *p* = 0.039, *η_p_*^2^ = 0.067), F (*F*(1, 62) = 5.80, *p* = 0.019, *η_p_*^2^ = 0.086) and occurrence of microstates D (*F*(1, 62) = 4.46, *p* = 0.039, *η_p_*^2^ = 0.067); however, these results should be interpreted with caution given that the original effects did not survive FDR correction.

### Comparison of baseline microstate transition probabilities between the NSSI and HC groups

As shown in **Supplementary Table 2**; **Figure 3D**, in uncorrected exploratory analyses, the NSSI group showed nominally significant differences in two transition probabilities relative to the HC group. Specifically, the transition probability from microstate A to B was higher in the NSSI group (*t*_65_ = 2.14, *p* = 0.036, *p*_FDR_ = 0.337, Cohen’s *d* = 0.52), as was the transition probability from microstate F to D (*t*_65_ = 2.32, *p* = 0.023, *p*_FDR_ = 0.337 Cohen’s *d* = 0.56). No other transition probabilities differed significantly between groups.

As sensitivity analyses for these nominal exploratory findings, we performed ANCOVAs with age, gender, and education as covariates. The results were consistent with the primary analyses (A to B: *F*(1, 62) = 4.66, *p* = 0.035, *η_p_*^2^ = 0.070; F to D: *F*(1, 62) = 5.14, *p* = 0.027, *η_p_*^2^ = 0.077); however, these ANCOVA results should be interpreted cautiously given that the original transition probability differences did not survive FDR correction.

### Correlation analyses with psychological variables

There were no statistically significant associations between HAMD, HAMA, and microstate dynamics (all *p*s > 0.20).

### Exploratory multivariate pattern analysis

Among the 35 baseline microstate features, the top 10 were first selected according to Relief-F importance rankings (**Supplementary Figure 1**). In addition, six features showed statistically significant between-group differences (*p* < 0.05). After merging and removing duplicates, a core feature set of 11 variables was retained, including durations of microstates A, B, and F; occurrences of microstates B and D; coverage of microstate B; and transition probabilities A→B, A→C, D→B, F→C, and F→D.

Using this core feature set, the SVM classifier achieved a mean accuracy of 88.02% across the five outer folds under nested cross-validation, with a mean precision of 84.64%, a mean recall of 94.28%, and a mean F1-score of 89.12% (**Supplementary Table 3**). ROC analysis yielded a mean AUC of 94.58% (**Supplementary Figure 2**). Permutation testing (1000 iterations) produced a null distribution with a mean accuracy of 51.3% ± 4.4% and a permutation p-value = 0.001, indicating that the observed classification performance was significantly above chance. Because feature rankings may be unstable in modest samples and no independent external validation sample was available, these classification results should be interpreted as exploratory and hypothesis-generating. The retained feature set is best understood as a distributed multivariate profile containing group-related information, rather than as evidence for a clinically validated biomarker.

### Effects of acute social exclusion on microstate duration, occurrence, and coverage in adolescents with NSSI

Post-task verbal reports indicated that participants in the acute social exclusion condition generally described feelings of being ignored, excluded, or distressed during the Cyberball task, supporting the intended direction of the manipulation. Because these responses were not collected using a standardized quantitative measure, they were not subjected to formal statistical analysis.

**Figure 4:**
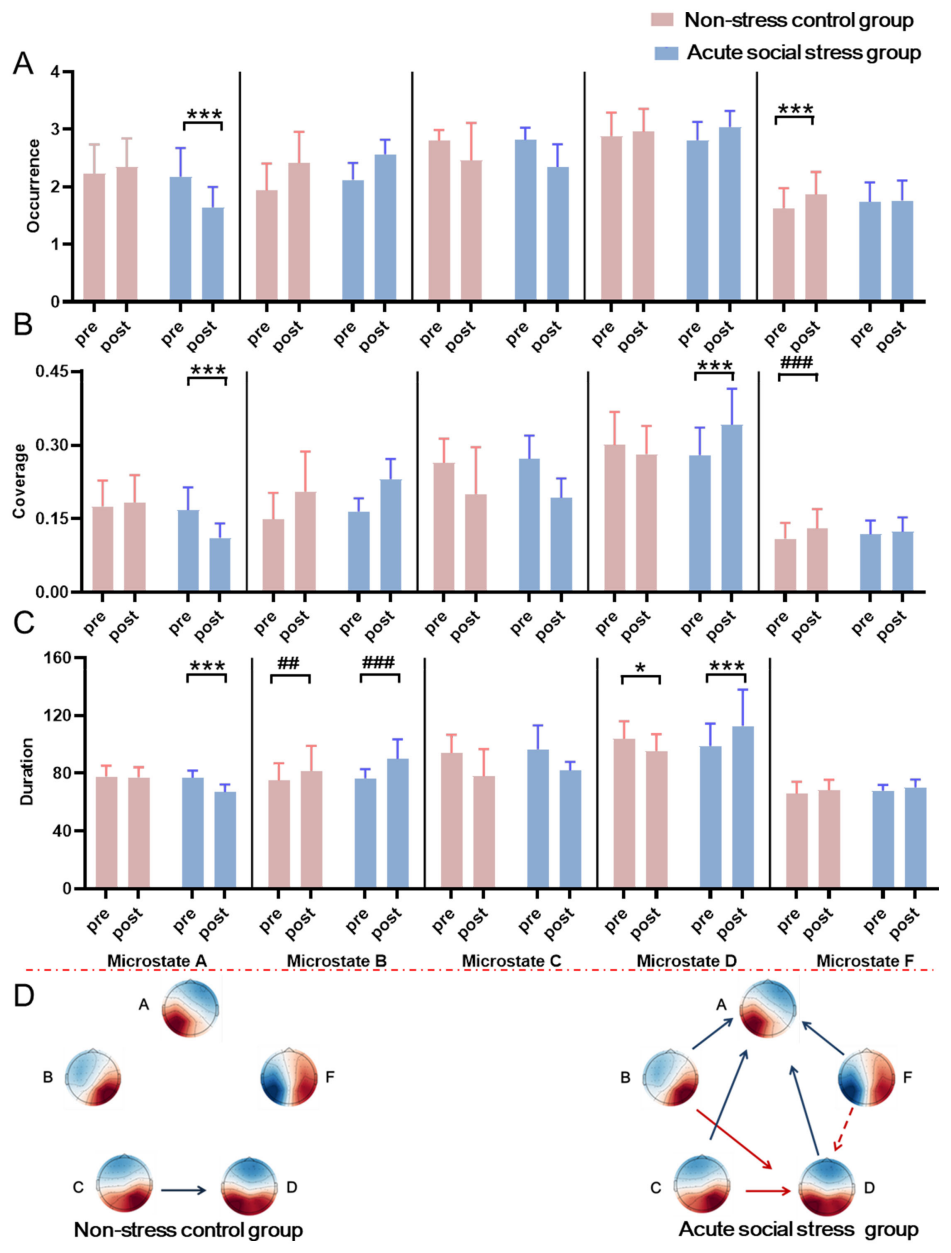
Interaction effects of acute social exclusion and time on microstate parameters in adolescents with NSSI. Mean values and standard deviations are shown for the non-stress control group (pink, n = 13) and the acute social exclusion group (blue, n = 19). **(A)** Occurrence rate of microstate classes A–D and F. **(B)** Time coverage of microstate classes A–D and F. **(C)** Mean duration of microstate classes A–D and F. **(D)** Changes in transition probabilities after the Cyberball task. Blue arrows indicate decreases, and red arrows indicate increases after Cyberball; red dashed arrows indicate interaction effects that did not survive FDR correction. In panels **A–C**, markers denote group-by-time interactions. ** and *** indicate interactions that survived FDR correction, with Bonferroni correction applied to *post hoc* comparisons (*p* < 0.01 and *p* < 0.001, respectively). **##** and **###** indicate interactions that did not survive FDR correction; exploratory *post hoc* comparisons were Bonferroni-corrected (*p* < 0.01 and *p* < 0.001, respectively).

A significant group-by-time interaction was observed for the occurrence of microstates A (*F*(1,30) = 27.71, *p* < 0.001, *p*_FDR_ < 0.001, *η_p_*^2^ = 0.480) and F (*F*(1,30) = 9.06, *p* = 0.005, *p*_FDR_ = 0.013 *η_p_*^2^ = 0.232) (**Figure 4A**; **Supplementary Table 4**). *Post hoc* analyses showed that the Cyberball task significantly reduced the occurrence of microstate A in the acute social exclusion group (*p*_Bonferroni_ *<*0.001, *η_p_*^2^ = 0.609), but not in the non-stress control group (*p*_Bonferroni_ = 0.249, *η*_p_^2^ = 0.044). In contrast, occurrence of microstate F increased significantly in the non-stress control group (*p*_Bonferroni_ < 0.001, *η_p_*^2^ = 0.384), but did not change in the acute social exclusion group (*p*_Bonferroni_ = 0.618, *η_p_*^2^ = 0.008).

Significant group-by-time interactions were also found for the coverage of microstates A (*F*(1,30) = 21.39, *p* < 0.001, *p*_FDR_ < 0.001, *η_p_*^2^ = 0.302), D (*F*(1,30) = 12.62, *p* = 0.001, *p*_FDR_ = 0.003, *η_p_*^2^ = 0.296), and F (*F*(1,30) = 5.33, *p* = 0.028, *p*_FDR_ = 0.06, *η_p_*^2^ = 0.151) (**Figure 4B**; **Supplementary Table 4**). The interaction for microstate F coverage did not survive FDR correction and should be regarded as a nominal finding. *Post hoc* analyses indicated that the Cyberball task reduced coverage of microstate A in the acute social exclusion group (*p*_Bonferroni_ < 0.001, *η_p_*^2^ = 0.581), but not in the non-stress control group (*p*_Bonferroni_ = 0.511, *η_p_*^2^ = 0.015). Coverage of microstate D increased in the acute social exclusion group (*p*_Bonferroni_ < 0.001, *η_p_*^2^ = 0.385), but not in the non-stress control group (*p*_Bonferroni_ = 0.312, *η_p_*^2^ = 0.034). Coverage of microstate F increased in the non-stress control group (*p*_Bonferroni_ < 0.001, *η_p_*^2^ = 0.385), but not in the acute social exclusion group (*p*_Bonferroni_ = 0.202, *η_p_*^2^ = 0.054).

In addition, significant group-by-time interactions were observed for the duration of microstates A (*F*(1,30) = 16.03, *p* < 0.001, *p*_FDR_ < 0.001, *η_p_*^2^ = 0.348), B (*F*(1,30) = 4.90, *p* = 0.035, *p*_FDR_ = 0.066, *η_p_*^2^ = 0.140), and D (*F*(1,30) = 19.59, *p* < 0.001, *p*_FDR_ < 0.001, *η_p_*^2^ = 0.395) (**Figure 4C**; **Supplementary Table 4**). The interaction for microstate B duration did not survive FDR correction and should be regarded as a nominal finding. *Post hoc* analyses showed that duration of microstate A decreased significantly after Cyberball in the acute social exclusion group (*p*_Bonferroni_ < 0.001, *η_p_*^2^ = 0.348), but not in the non-stress control group (*p*_Bonferroni_ = 0.770, *η_p_*^2^ = 0.003). Duration of microstate B increased in both groups (non-stress control group: *p*_Bonferroni_ = 0.009, *η_p_*^2^ = 0.204; acute social exclusion group: *p*_Bonferroni_ < 0.001, *η_p_*^2^ = 0.608). Duration of microstate D increased in the acute social exclusion group (*p*_Bonferroni_ < 0.001, *η_p_*^2^ = 0.382), but decreased in the non-stress control group (*p*_Bonferroni_ = 0.037, *η_p_*^2^ = 0.137).

### Effects of acute social exclusion on microstate transition probabilities in adolescents with NSSI

**Figure 4D**; **Supplementary Table 5** summarize the effects of the group-by-time interaction on microstate transition probabilities. In the acute social exclusion group, Cyberball significantly increased transitions from microstates B (*p*_Bonferroni_ < 0.001, *η_p_*^2^ = 0.557), C (*p*_Bonferroni_ = 0.008, *η_p_*^2^ = 0.211), and F (*p*_Bonferroni_ = 0.001, *η_p_*^2^ = 0.336) to microstate D. The interaction for the transition probability F→D did not survive FDR correction and should be regarded as a nominal finding. At the same time, the acute social exclusion group showed significantly decreased transitions from microstates B (*p*_Bonferroni_ < 0.001, *η_p_*^2^ = 0.474), C (*p*_Bonferroni_ < 0.001, *η_p_*^2^ = 0.635), D (*p*_Bonferroni_ < 0.001, *η_p_*^2^ = 0.385), and F (*p*_Bonferroni_ < 0.001, *η_p_*^2^ = 0.499) to microstate A.

In the non-stress control group, the only significant change was a decrease in the transition probability from microstate C to D (*p*_Bonferroni_ = 0.028, *η_p_*^2^ = 0.151). Overall, these results indicate that acute social exclusion was associated with a marked reorganization of transition dynamics toward the attention-related microstate D in adolescents with NSSI.

### Bayesian robustness analysis

Bayesian repeated measures ANOVA results were similar to the primary findings. Detailed results are provided in the **Supplementary Materials**.

## Discussion

The present study examined resting-state EEG microstate dynamics and their state-dependent changes after acute social exclusion in a clinically recruited, medicated, inpatient adolescent NSSI sample. Three main findings emerged. First, after multiple-comparison correction, adolescents with NSSI showed a robustly reduced duration of microstate A at baseline. Exploratory analyses further suggested a shorter duration of microstates B and F, higher occurrence of microstate D, and altered transition probabilities A→B and F→D. Second, a multivariate pattern analysis suggested that distributed microstate features contained group-related information, though this requires independent replication. Third, within the NSSI group, acute social exclusion was followed by a reorganization of microstate dynamics characterized by reduced microstate A and increased microstate D expression, and a broader shift in transition probabilities toward D. Because the sample was medicated and no MDD-only clinical-control group was included, these findings should be interpreted as microstate alterations observed in this specific clinical context, with uncertain specificity to NSSI.

### Altered resting-state microstate dynamics in NSSI

Before discussing potential functional interpretations, it is important to emphasize the interpretive boundaries of these baseline findings. Only the reduced duration of microstate A survived correction for multiple comparisons; all other baseline group differences—including durations of B and F, occurrence of D, and transition probabilities—did not remain significant after correction and should be considered exploratory. Furthermore, all NSSI participants were hospitalized and receiving psychotropic medication, and the study lacked a clinical-control group. Therefore, any functional interpretations below are offered as tentative, hypothesis-generating observations that require replication in independent, unmedicated, and multi-group samples.

The core corrected finding was reduced duration of microstate A, which has been linked to auditory-phonological processing (18, 21, 48). This may point to altered intrinsic organization of sensory processing in this clinical sample. In exploratory analyses, adolescents with NSSI also exhibited shorter durations of microstates B and F. Microstate B has been associated with visual processing, and microstate F, topographically similar to microstate E in prior studies, has been linked to self-referential processing and the default mode network (DMN) (49, 50). If replicated, these patterns would be consistent with reduced stability of self-related processing. This was accompanied, at a nominal level, by higher occurrence of microstate D, associated with the dorsal attention network (22, 51). Reduced temporal stability of these states may therefore suggest altered intrinsic organization of sensory and internally oriented processing. One speculative interpretation is that this sample showed greater recruitment of attention-related processing even during rest.

Transition dynamics, though not surviving correction, provide supplementary information. The nominally increased F→D transition probability raises the possibility of rapid shifts from self-referential toward attention-related states. However, this remains a tentative observation that requires confirmation in future studies.

The multivariate pattern analysis showed that, in this sample, distributed microstate features—rather than any single parameter—carried information that separated the NSSI and HC groups. The retained feature set included durations of A, B, and F; occurrences of B and D; coverage of B; and transitions involving A, B, D, and F. This distributed pattern is broadly compatible with models of NSSI emphasizing emotion dysregulation and heightened sensitivity to interpersonal cues. However, given the modest sample, absence of independent validation, and lack of a clinical-control group, these classification results should be interpreted as an exploratory multivariate description of group-related information, rather than as evidence for a clinically validated biomarker.

An important interpretive issue concerns the overlap between NSSI and depressive psychopathology, and the potential influence of medication and hospitalization. Prior EEG microstate studies have attempted to disentangle these factors using multi-group designs. Hu et al. (2023) reported that MDD-only adolescents showed higher microstate A contribution and occurrence than both HC and NSSI groups, whereas adolescents with NSSI showed increased B→C transition probability relative to HCs (34). Song et al. (2025) reported reduced microstate B activity and lower D→B transition probability in adolescents with MDD+NSSI, and showed that classification between MDD+NSSI and MDD-only groups was more modest than between MDD+NSSI and HCs (28). Together, these studies suggest that NSSI-related microstate findings partly overlap with depression-related alterations, and the two are not fully separable with current designs.

In our sample, HAMD and HAMA scores were not significantly correlated with the microstate metrics that differed between NSSI and HCs, suggesting that variation in depressive or anxiety symptom severity within the NSSI group did not linearly drive these effects. However, this analysis cannot rule out the influence of categorical depression status, medication, or broader transdiagnostic emotion-dysregulation mechanisms. Because the present study did not include an MDD-only clinical-control group, the findings should be interpreted as NSSI-associated microstate alterations in a clinically recruited adolescent sample, rather than as definitive evidence of NSSI-specific neurophysiology.

### Neurophysiological reorganization following acute social exclusion in NSSI

The social exclusion manipulation provided further evidence that within this NSSI sample, acute interpersonal stress was followed by a reorganization of large-scale brain dynamics. Following Cyberball-induced exclusion, the exclusion subgroup showed reduced microstate A and increased microstate D expression relative to the non-stress subgroup. This pattern may suggest reduced sensory-perceptual stability together with enhanced recruitment of attention-related processing under conditions of social rejection. The post-task verbal debriefing indicated that participants in the exclusion condition generally perceived the task as subjectively distressing, providing qualitative face-valid evidence for the manipulation. However, standardized quantitative measures of exclusion-related distress were not administered, and therefore the magnitude of induced social exclusion was not formally quantified.

This interpretation is broadly consistent with prior neuroimaging findings showing altered recruitment of prefrontal systems during social exclusion in adolescents with NSSI (52). These regions are implicated in attentional control, monitoring, and cognitive appraisal, which may accord with the increased expression of microstate D observed here. Importantly, transition analyses showed that multiple microstate classes, including B, C, and F, shifted more strongly toward microstate D after exclusion, whereas transitions toward microstate A were reduced. This pattern suggests a broader reorganization of large-scale brain dynamics toward attention-related processing following interpersonal stress.

Such a shift, if replicated, would be compatible with the hypothesis that interpersonal stress engages attention-related networks more strongly in individuals with NSSI (53). but this remains to be tested against clinical and healthy comparison groups. Rather than maintaining a balanced resting-state configuration, the brain may become more strongly biased toward vigilance-related processing after social exclusion. This may help explain why social rejection is experienced as particularly distressing by vulnerable adolescents, though whether such neurophysiological shifts directly precede self-injurious urges remains to be tested in studies with real-time urge assessment.

By contrast, the non-stress subgroup showed a comparatively less pronounced pattern of reconfiguration, including increased prominence of microstate F and reduced transition from microstate C to D. This may suggest that, in the absence of exclusion-related distress, participants were better able to maintain or restore a more stable resting-state configuration. Critically, because healthy controls did not undergo the same pre-post Cyberball paradigm, the present design cannot determine whether the observed neurophysiological shift toward microstate D is specific to NSSI or represents a general adolescent response to social exclusion. The findings should therefore be interpreted as state-dependent reorganization within an NSSI sample, rather than as evidence for NSSI-specific stress reactivity.

### Limitations and future directions

Several limitations should be acknowledged. First, the comparison between the NSSI and HC groups was cross-sectional, which limits causal inference. Longitudinal studies are needed to determine whether the observed microstate patterns precede the onset of NSSI or change with symptom progression. Second, although the sample size is comparable to that of many EEG studies, replication in larger and more diverse samples is necessary, particularly for the machine learning analysis. The present classification results should therefore be interpreted as preliminary and in need of external validation. Future studies may also examine whether these microstate features change with treatment. If so, EEG microstate dynamics may prove useful not only as markers of NSSI vulnerability but also as potential indicators of treatment response in interventions targeting emotion regulation and interpersonal stress sensitivity. Third, the relatively modest GEV of approximately 63% should be regarded as a methodological limitation. This value is lower than those commonly reported in many adult resting-state EEG microstate studies, and it indicates that a considerable portion of the EEG signal variance was not captured by the four canonical microstate maps. This may reflect several features of the present dataset and analysis pipeline, including the eyes-open recording condition, the adolescent psychiatric inpatient sample, strict artifact rejection, and the 2–20 Hz bandpass filter chosen to minimize electromyographic contamination. Importantly, GEV values were highly similar across the HC group (63.07%), the NSSI group (63.52%), and the post-task NSSI subgroups (63.48% and 63.90%), suggesting that the main group and group-by-time effects were unlikely to be driven solely by systematic differences in model fit. Nevertheless, given the modest GEV, the present findings may depend on the selected preprocessing and clustering pipeline. Future studies should examine whether these results replicate under alternative preprocessing bands, microstate solutions, and independent samples. Fourth, the clinical characterization of NSSI was limited. The inclusion criterion required at least five days of intentional self-injury during the past year, but standardized measures of NSSI frequency, recency, severity, functions, urges, and duration of NSSI history were not systematically collected. Consequently, we could not examine potential clinical heterogeneity within the NSSI group or its relationship to microstate dynamics. Furthermore, although participants were randomly assigned to the Cyberball conditions, we cannot rule out the possibility that the post-manipulation subgroups differed in unmeasured NSSI severity, which might partly account for the observed post-task differences rather than the experimental manipulation itself. This limited phenotyping also restricts the interpretation of baseline group differences, as we cannot determine which specific dimensions of NSSI psychopathology the observed microstate alterations are most closely associated with. Future studies should incorporate validated multi-dimensional NSSI assessment tools to characterize the clinical phenotype more comprehensively and to examine its potential influence on both resting-state and task-related EEG microstate dynamics. Fifth, all NSSI participants were receiving inpatient pharmacological treatment, but detailed medication records were unavailable. Given that psychotropic medications can influence EEG microstate parameters, we cannot rule out the possibility that some of the observed group differences reflect treatment-related neurophysiological effects rather than, or in addition to, NSSI psychopathology. This directly limits the specificity of our findings. Future studies should aim to include medication-naive patients or, at minimum, systematically record and control for medication class. Sixth, the Cyberball manipulation check relied on qualitative verbal debriefing rather than on standardized quantitative instruments. Consequently, the magnitude of subjectively experienced social exclusion and distress could not be formally verified, which limits confidence in the experimental manipulation and tempers interpretation of the post-task EEG findings. Future studies should incorporate validated quantitative manipulation-check measures. Seventh, another important limitation concerns depressive psychopathology. HAMD and HAMA scores were available only in the NSSI group, and the study did not include an MDD-only clinical-control group. Therefore, although within-group correlation analyses suggested that depressive and anxiety symptom severity did not linearly account for the observed microstate differences, the present design cannot determine whether these findings are specific to NSSI or reflect depression-related or transdiagnostic mechanisms. Future studies should include MDD-only, MDD+NSSI, and HC groups with comparable symptom assessments across all groups. Finally, the 2–20 Hz bandpass filter was chosen *a priori* based on established conventions in the EEG microstate literature to isolate canonical microstates while reducing high-frequency muscle artifact, which is particularly problematic in resting-state recordings from adolescent clinical populations. However, this filter precludes the examination of high-beta and gamma band microstates. Given that high-frequency oscillations have been linked to anxiety and hyperarousal—features common in NSSI—the present findings may not capture microstate dynamics above 20 Hz that could be relevant to these clinical dimensions. Future studies employing advanced artifact rejection techniques and larger samples should systematically examine whether the observed microstate alterations are robust across alternative filter bands and whether additional alterations emerge in higher-frequency microstates.

## Conclusion

This study identified EEG microstate alterations in a clinically recruited, medicated adolescent inpatient NSSI sample. The strongest corrected baseline finding was reduced microstate A duration; additional microstate and transition findings should be treated as exploratory. Following the Cyberball paradigm, the exclusion subgroup showed a reorganization of brain dynamics involving reduced A and increased D expression, although the subjective impact of the manipulation was not quantitatively validated. These findings suggest that EEG microstates may provide candidate indices for studying large-scale network dynamics in this population. However, the specificity of these patterns to NSSI remains unresolved given the absence of an MDD-only clinical-control group, the potential influence of medication, and the lack of healthy Cyberball comparison. Therefore, these results should be regarded as preliminary and descriptive of a specific clinical sample. Replication in multi-group designs including medication-naive NSSI, MDD-only, and MDD+NSSI groups is essential.

## Data Availability

The raw data supporting the conclusions of this article will be made available by the authors, without undue reservation.
