# Supplementary material for: Altered EEG microstate dynamics in adolescents with non-suicidal self-injury at rest and following acute social exclusion
Source: Front Psychiatry. 2026 Jul 16;17:1853860. doi: 10.3389/fpsyt.2026.1853860 (PMC13422403; doi:10.3389/fpsyt.2026.1853860)
Supplement: Supplementary file 1 [file DataSheet1.pdf]

Supplementary Material

1 Supplementary Method

1.1 SVM model

Model construction and performance evaluation adopted a nested cross validation strategy to avoid information “leak” into the model and overfit the data. The outer loop was set to 5-fold stratified cross-validation for unbiased performance estimation, while the inner loop was set to 3-fold stratified cross-validation to optimize the hyperparameters of the SVM model using only the training set from the outer loop. Specifically, a Radial Basis Function (RBF) kernel was employed to capture potential non-linear decision boundaries between NSSI and HC groups. Within each inner loop, a grid search was conducted over the regularization parameter C:{0.01, 0.1, 1, 5} and the kernel coefficient γ: {0.005, 0.01, 0.05}. The optimal hyperparameter combination was selected based on the highest mean balanced accuracy across the 3 inner folds, which accounts for potential class imbalance in our sample. To eliminate errors induced by numerical discrepancies among features, a standardscaler was used to perform Z-score standardization on the features. In the outer loop, the standardscaler was only fitted on the training set, and applied to both the training and test sets to prevent information leakage from the test set to model.

Given the limited sample size in this study, a permutation test was conducted to exclude random chance and sampling bias and validate the statistical significance of the SVM classification performance. Permutation test adopted the same hyperparameters and cross-validation strategy as the original model. We randomly shuffled the training set class labels for 1000 iterations while keeping the feature data unchanged. The SVM model was retrained in each iteration to generate a null distribution. The true classification accuracy of the original model was compared with the null distribution to compute the p-value. Statistical significance was defined as $p < 0.05$.

1.2 Bayesian robustness analysis

For the analytical robustness, a Bayesian repeated measures ANOVA approach was used to provide probabilistic statements of the acute social stress effect for the dependent variable which show the significant interaction effect in the mixed ANOVA analyses. Bayes Factor (BF_{10}) were reported as a relative measure of evidence (compared with the null model).The alternative hypothesis (H1) was supported when the BF > 3, while the null hypothesis (H0) was favored when BF < 1/3. Interpretations of BF_{10} followed the established evidence categories: weak evidence for H1 ($1 < BF_{10} < 3$; positive evidence ($BF_{10} \geq 3$), strong evidence ($BF_{10} \geq 10$), very strong evidence ($BF_{10} \geq 100$)(1). For the sake of clarity, we restricted our reporting to the best-supported model, unless competing models yielded comparable fit to the data, as determined by pairwise comparisons with the top-performing model. Furthermore, we implemented effect-level analyses that compared models including a given effect (interaction) to structurally equivalent models from which the effect was omitted, using the inclusion Bayes factor (BF_{incl}) to provide a relative assessment of evidence in favor of including the factor.

2 Supplementary Result

2.1 Permutation test

The original SVM model had a mean accuracy of 88%. The 1000 permuted null models yielded a near-random performance with a mean accuracy of 51.3% ±4.4%. *p*-value was 0.001, which was significantly lower than the 0.05 threshold. This result confirmed that the high classification accuracy was not caused by random sampling error, and the model exhibited above-chance separability in this sample.

2.2 Bayesian robustness analysis

For the duration of microstate A, Bayesian repeated-measures ANOVA revealed that the best-supported model was the full model with the interaction term ($P_{M|data}$) = 0.987, BF_{10} = 1.95e + 5). There was decisive evidence for timepoint(BF_{incl} = 2.41e + 4), acute social stress (BF_{incl} = 422.9), and positive evidence for the acute social stress×timepoint interaction (BF_{incl} = 310.2).

For the duration of microstate B, the best-supported model was the full model with the interaction term ($P_{M|data}$) = 0.467, BF_{10} = 6.39e + 4). There was decisive evidence for timepoint(BF_{incl} = 5.69e + 4), weak evidence for acute social stress (BF_{incl} = 1.44), and positive evidence for the acute social stress×timepoint interaction (BF_{incl} = 3.5).

For the duration of microstate D, the best-supported model was the full model with the interaction term ($P_{M|data}$) = 0.969, BF_{10} = 81.89). There was strong evidence for timepoint(BF_{incl} = 34.96), and acute social stress (BF_{incl} = 33.29), and decisive evidence for the acute social stress×timepoint interaction (BF_{incl} = 124.7).

For the occurrence of microstate A, the best-supported model was the full model with the interaction term ($P_{M|data}$) = 0.999, BF_{10} = 5.24e + 4). There was decisive evidence for timepoint(BF_{incl} = 5832), acute social stress (BF_{incl} = 2344) and the acute social stress × timepoint interaction (BF_{incl} = 3204).

For the occurrence of microstate F, the best-supported model was the full model with the interaction term ($P_{M|data}$) = 0.671, BF_{10} = 18). There was strong evidence for timepoint (BF_{incl} = 11.44), weak evidence for acute social stress (BF_{incl} = 2.46), and positive evidence for the acute social stress×timepoint interaction (BF_{incl} = 8.14).

For the coverage of microstate A, the best-supported model was the full model with the interaction term ($P_{M|data}$) = 0.995, BF_{10} = 2.45e + 4). There was decisive evidenc for timepoint(BF_{incl} = 3073), acute social stress (BF_{incl} = 755.3), and acute social stress×timepoint interaction (BF_{incl} = 794.7).

For the coverage of microstate D, the best-supported model was the full model with the interaction term ($P_{M|data}$) = 0.879, BF_{10} = 33.13). There was strong evidence for timepoint(BF_{incl} = 16.20), and acute social stress×timepoint interaction (BF_{incl} = 28.94), and weak evidence for acute social stress (BF_{incl} = 1.44), and positive evidence for acute social stress (BF_{incl} = 7.65).

For the coverage of microstate F, the best-supported model was the full model with the interaction term (PM|data) = 0.879, BF_{10} = 33.13). But there was weak evidence for acute social stress×timepoint interaction (BFincl = 2.73), and acute social stress (BFincl = 1.08).

3 Supplementary Figures and Tables

3.1 Supplementary Figures

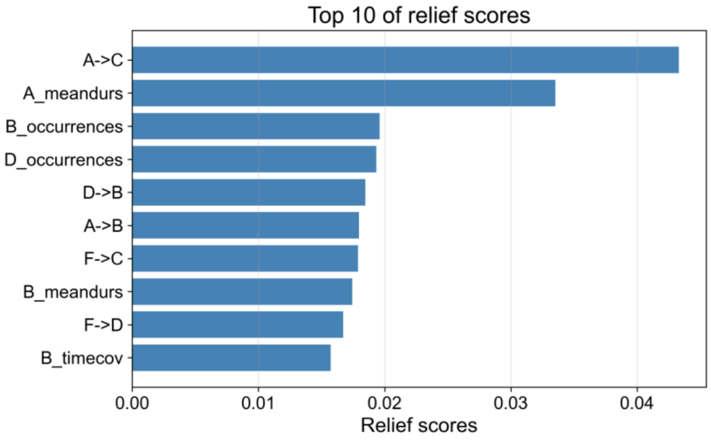

Supplementary Figure 1. The weight scores of top 10 features for the Relief-F model.

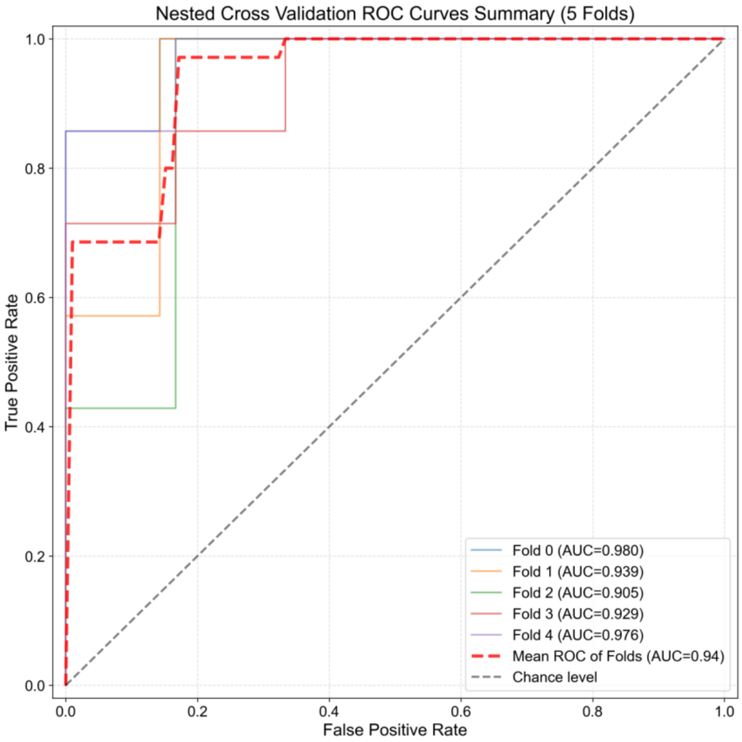

Supplementary Figure 2. The ROC curve for the exploratory multivariate pattern analysis separating adolescents with NSSI from HCs using the 11 retained microstate features. AUC = area under the curve.

3.2 Supplementary Table

Supplementary Table 1. Comparison of baseline EEG microstate parameters between the NSSI and HC groups

	HC Mean(SD)	NSSI Mean(SD)	*t* value	*p* value	p_{FDR}
Duration					
A	87.57(15.53)	77.93(6.68)	3.35**	0.001	0.015
B	81.45(10.09)	75.80(9.10)	2.41*	0.019	0.131
C	96.40(20.84)	95.32(15.84)	0.24	0.810	0.868
D	104.34(22.04)	101.15(14.75)	0.70	0.485	0.808
F	71.62(7.04)	67.72(7.23)	2.23*	0.029	0.131
Occurrence					
A	2.19(0.45)	2.22(0.51)	0.26	0.796	0.868
B	1.90(0.44)	2.03(0.37)	1.40	0.166	0.311
C	2.60(0.47)	2.78 (0.23)	1.97	0.053	0.159
D	2.64(0.36)	2.83(0.37)	2.15*	0.035	0.131
F	1.58(0.38)	1.71(0.34)	1.44	0.154	0.311
Coverage					
A	0.20(0.07)	0.18 (0.05)	1.40	0.166	0.311
B	0.16(0.05)	0.16 (0.04)	0.06	0.950	0.950
C	0.26(0.09)	0.27 (0.05)	0.56	0.581	0.811
D	0.28(0.08)	0.29(0.06)	0.53	0.595	0.811
F	0.11(0.03)	0.12(0.03)	0.36	0.721	0.868

*$p < .05$, **$p < .01$.

Supplementary Table 2. Comparison of baseline transition probabilities between the NSSI and HC groups

	HC Mean(SD)	NSSI Mean(SD)	*t* value	*p* value	p_{FDR} value
A→ B	0.182(0.040)	0.202(0.037)	2.144*	0.036	0.337
A→ C	0.323(0.084)	0.296(0.033)	1.712	0.095	0.337
A→ D	0.327(0.075)	0.342(0.064)	0.899	0.372	0.620
A→ F	0.168(0.044)	0.160(0.035)	0.841	0.403	0.620
B→ A	0.216(0.060)	0.222(0.050)	0.437	0.663	0.780
B→ C	0.294(0.072)	0.317(0.067)	1.312	0.194	0.400
B→ D	0.317(0.068)	0.298(0.045)	1.369	0.176	0.400
B→ F	0.172(0.042)	0.163(0.035)	0.955	0.343	0.620
C→ A	0.267(0.067)	0.244(0.055)	1.584	0.118	0.337
C→ B	0.208(0.047)	0.218 (0.059)	0.723	0.472	0.629
C→ D	0.360(0.073)	0.359(0.076)	0.060	0.952	0.952
C→ F	0.165(0.040)	0.181(0.033)	1.712	0.092	0.337
D→ A	0.266(0.076)	0.244 (0.065)	1.294	0.200	0.400
D→ B	0.221(0.065)	0.220 (0.040)	0.117	0.908	0.952
D→ C	0.348(0.081)	0.358 (0.070)	0.541	0.590	0.738
D→ F	0.164(0.037)	0.178 (0.032)	1.626	0.109	0.337
F→ A	0.250(0.064)	0.228(0.047)	1.633	0.107	0.337
F→ B	0.226(0.057)	0.215 (0.060)	0.769	0.444	0.629
F→ C	0.272(0.065)	0.276(0.030)	0.325	0.746	0.829
F→ D	0.252(0.056)	0.282(0.048)	2.323*	0.023	0.337

*$p < .05$.

Supplementary Table 3. Performance of the exploratory multivariate pattern analysis under nested cross-validation

Outer Fold	ACC	ACC 95%CI	AUC	AUC 95% CI	Precision	Recall	F1
1	85.70%	0.57~1.0	98%	0.82~1.0	85.70%	85.70%	85.70%
2	92.90%	0.71~1.0	93.90%	0.67~1.0	87.50%	100%	93.30%
3	92.30%	0.58~1.0	90.50%	0.65~1.0	87.50%	100%	93.30%
4	76.90%	0.66~0.92	92.90%	0.67~1.0	75.00%	85.70%	80.00%
5	92.30%	0.62~1.0	97.60%	0.71~1.0	87.50%	100%	93.30%
Mean	88.02%	-	94.58%	-	84.64%	94.28%	89.12%

Supplementary Table 4. The interaction effect of acute social stress and group on microstate dynamics in adolescents with NSSI

	Non-stress control group		Acute social stress group		*F* value	*p* value	p_{FDR} value	Post-hoc comparisons
	pre Mean (SD)	post Mean (SD)	pre Mean (SD)	post Mean (SD)				
Duration								
A	77.70 (7.60)	77.17 (6.99)	76.84 (4.95)	66.95 (5.23)	16.03	< 0.001	< 0.001	$pre_{ASG} > post_{ASG}$
B	75.00 (11.87)	81.65 (17.23)	76.45 (6.45)	89.98 (13.51)	4.90	0.035	0.066	$Pre_{NCG} < post_{NCG}$ $Pre_{ASG} < post_{ASG}$
C	93.97 (12.63)	77.96 (18.82)	96.54 (16.53)	81.92 (5.84)	0.04	0.85	0.911	
D	103.81 (12.07)	95.39 (11.71)	89.91 (15.61)	112.67 (25.58)	19.59**	<0.001	< 0.001	$Pre_{NCG} > post_{NCG}$ $Pre_{ASG} < post_{ASG}$
F	65.82 (8.31)	68.15 (7.38)	67.76 (4.22)	70.06 (5.58)	0.001	0.982	0.982	
Occurrence								
A	2.23 (0.50	2.35 (0.50)	2.18 (0.50)	1.64 (0.36)	27.71***	<0.001	< 0.001	$pre_{ASG} > post_{ASG}$
B	1.94 (0.47)	2.42 (0.54)	2.12 (0.29)	2.56 (0.26)	0.23	0.64	0.796	
C	2.80 (0.18)	2.46 (0.65)	2.81 (0.21)	2.34 (0.40)	0.39	0.54	0.736	

D	2.88 (0.41)	2.96 (0.39)	2.81 (0.32)	3.04 (0.28)	1.67	0.21	0.350	
F	1.63 (0.35)	1.87 (0.39)	1.74 (0.34)	1.76 (0.35)	9.06**	0.005	0.013	$Pre_{NCG} < post_{NCG}$
Coverage								
A	0.18 (0.05)	0.18 (0.06)	0.17 (0.05)	0.11 (0.03)	21.39***	< 0.001	< 0.001	$pre_{ASG} > post_{ASG}$
B	0.15 (0.05)	0.20 (0.08)	0.16 (0.03)	0.23 (0.04)	1.19	0.28	0.420	
C	0.26 (0.05)	0.20 (0.10)	0.27 (0.05)	0.19 (0.04)	0.17	0.69	0.796	
D	0.30 (0.07)	0.28 (0.06)	0.28 (0.06)	0.34 (0.07)	12.62**	0.001	0.003	$pre_{ASG} < post_{ASG}$
F	0.11 (0.03)	0.13 (0.04)	0.12 (0.03)	0.12 (0.03)	5.33*	0.028	0.06	$Pre_{NCG} < post_{NCG}$

ASG: Acute social stress group; NCG: Non-stress control group *p < .05; **p < .01; ***p < .001

Supplementary Table 5. The interaction effect of acute social stress and group on transition probability in adolescents with NSSI

	Non-stress control group		Acute social stress group		F value	p value	p_{FDR} value	Post-hoc comparisons
	pre Mean (SD)	post Mean (SD)	pre Mean (SD)	post Mean (SD)				
A→B	0.20 (0.05)	0.24 (0.06)	0.21 (0.03)	0.26 (0.03)	1.02	0.321	0.569	
A→C	0.30 (0.04)	0.26 (0.11)	0.30 (0.03)	0.25 (0.03)	0.085	0.772	0.908	
A→D	0.35 (0.07)	0.33 (0.06)	0.33 (0.07)	0.32 (0.04)	0.388	0.538	0.717	
A→F	0.15 (0.04)	0.17 (0.04)	0.16 (0.03)	0.17 (0.04)	2.70	0.111	0.247	
B→A	0.22 (0.05)	0.23 (0.04)	0.22 (0.04)	0.17 (0.04)	16.89***	<0.001	0.001	$Pre_{ASG} > post_{ASG}$
B→C	0.32 (0.07)	0.25 (0.08)	0.32 (0.06)	0.25 (0.04)	0.003	0.956	0.983	
B→D	0.30 (0.06)	0.33 (0.07)	0.29 (0.04)	0.40 (0.08)	9.24**	0.005	0.024	$Pre_{ASG} < post_{ASG}$
B→F	0.15 (0.04)	0.18 (0.05)	0.17 (0.03)	0.18 (0.04)	2.91	0.098	0.245	
C→A	0.25 (0.06)	0.24 (0.08)	0.24 (0.05)	0.15 (0.04)	18.52***	<0.001	0.001	$Pre_{ASG} > post_{ASG}$
C→B	0.21	0.25	0.23	0.28	0.70	0.409	0.604	

	(0.08)	(0.07)	(0.03)	(0.04)				
C→D	0.37<br>(0.08)	0.33<br>(0.04)	0.36<br>(0.08)	0.39<br>(0.08)	12.86**	0.001	0.007	Pre$_{NCG}$ > post$_{NCG}$<br>Pre$_{ASG}$ < post$_{ASG}$
C→F	0.18<br>(0.03)	0.18<br>(0.04)	0.18<br>(0.04)	0.18<br>(0.03)	0.94	0.341	0.568	
D→A	0.25<br>(0.08)	0.25<br>(0.06)	0.23<br>(0.06)	0.18<br>(0.03)	7.87**	0.009	0.03	Pre$_{ASG}$ > post$_{ASG}$
D→B	0.21<br>(0.05)	0.28<br>(0.09)	0.23<br>(0.03)	0.33<br>(0.06)	1.37	0.251	0.502	
D→C	0.37<br>(0.06)	0.27<br>(0.08)	0.36<br>(0.07)	0.28<br>(0.05)	0.27	0.609	0.761	
D→F	0.17<br>(0.03)	0.20<br>(0.05)	0.18<br>(0.03)	0.20<br>(0.03)	<0.001	0.983	0.983	
F→A	0.23<br>(0.04)	0.22<br>(0.05)	0.22<br>(0.05)	0.16<br>(0.03)	8.88**	0.006	0.024	Pre$_{ASG}$ > post$_{ASG}$
F→B	0.20<br>(0.07)	0.25<br>(0.08)	0.23<br>(0.05)	0.28<br>(0.06)	0.64	0.423	0.604	
F→C	0.27<br>(0.03)	0.23<br>(0.08)	0.28<br>(0.03)	0.24<br>(0.04)	0.04	0.837	0.930	
F→D	0.29<br>(0.05)	0.30<br>(0.05)	0.27<br>(0.04)	0.32<br>(0.04)	5.15*	0.031	0.09	Pre$_{ASG}$ < post$_{ASG}$

ASG: Acute social stress group; NCG: Non-stress control group *p < .05; **p < .01; ***p < .001
